# Epigallocatechin-3-gallate therapeutic potential in human diseases: molecular mechanisms and clinical studies

**DOI:** 10.1186/s43556-024-00240-9

**Published:** 2024-12-27

**Authors:** Manzar Alam, Mehak Gulzar, Mohammad Salman Akhtar, Summya Rashid, Zulfareen, Tanuja, Anas Shamsi, Md. Imtaiyaz Hassan

**Affiliations:** 1 Centre for Interdisciplinary Research in Basic Sciences, Jamia Millia Islamia, Jamia Nagar, New Delhi, 110025 India; 2 Department of Basic Medical Sciences, Faculty of Applied Medical Sciences, Albaha University, Albaha, Saudi Arabia; 3 Department of Pharmacology & Toxicology, College of Pharmacy, Prince Sattam Bin Abdulaziz University, PO Box 173, 11942 Al-Kharj, Saudi Arabia; 4 Center of Medical and Bio-Allied Health Sciences Research (CMBHSR), Ajman University, P.O. Box 346, Ajman, UAE

**Keywords:** Epigallocatechin gallate, Neurological disorders, Cancer therapy, Neuroinflammation, Targeted therapy, Natural products

## Abstract

Green tea has garnered increasing attention across age groups due to its numerous health benefits, largely attributed to Epigallocatechin 3-gallate (EGCG), its key polyphenol. EGCG exhibits a wide spectrum of biological activities, including antioxidant, anti-inflammatory, antibacterial, anticancer, and neuroprotective properties, as well as benefits for cardiovascular and oral health. This review provides a comprehensive overview of recent findings on the therapeutic potential of EGCG in various human diseases. Neuroprotective effects of EGCG include safeguarding neurons from damage and enhancing cognitive function, primarily through its antioxidant capacity to reduce reactive oxygen species (ROS) generated during physiological stress. Additionally, EGCG modulates key signaling pathways such as JAK/STAT, Delta-Notch, and TNF, all of which play critical roles in neuronal survival, growth, and function. Furthermore, EGCG is involved in regulating apoptosis and cell cycle progression, making it a promising candidate for the treatment of metabolic diseases, including cancer and diabetes. Despite its promising therapeutic potential, further clinical trials are essential to validate the efficacy and safety of EGCG and to optimize its delivery to target tissues. While many reviews have addressed the anticancer properties of EGCG, this review focuses on the molecular mechanisms and signaling pathways by which EGCG used in specific human diseases, particularly cancer, neurodegenerative and metabolic diseases. It serves as a valuable resource for researchers, clinicians, and healthcare professionals, revealing the potential of EGCG in managing neurodegenerative disorders, cancer, and metabolic diseases and highlighting its broader therapeutic values.

## Introduction

**Fig. 1:**
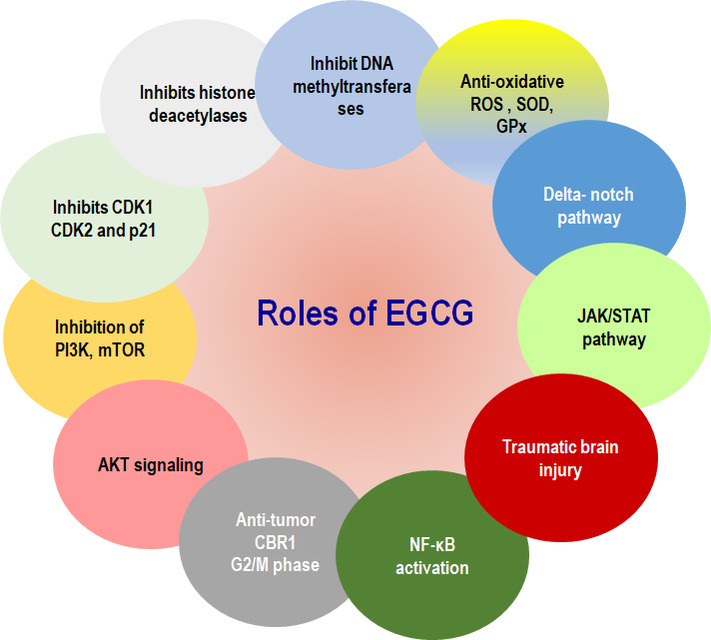
Mechanistic Overview of Modulation of Cellular Signaling Pathways by EGCG. This figure illustrates the multifaceted mechanism of EGCG and its interactions with key signaling and molecular pathways. EGCG is shown to regulate various target genes and proteins, including JAK/STAT, NF-κB, AKT, and Notch pathways, highlighting its critical roles in cellular processes such as apoptosis, proliferation, and survival. The diagram emphasizes the biological functions of EGCG, including its anti-cancer, anti-inflammatory, and antioxidant activities. By inhibiting oxidative stress, reducing inflammation, and promoting apoptosis in cancer cells, EGCG showcases its therapeutic potential in combating diseases like cancer, cardiovascular , and neurodegenerative diseases

Polyphenols are a group of bioactive compounds found in foods and beverages, mainly present in green tea. They act as an antioxidant, anti-inflammatory, and neuroprotective agent, which belongs to catechins [1]. Among many other compounds, the potential and major biochemical compound is Epigallocatechin-3-gallate (EGCG), the most abundant flavone-3-ol polyphenol present in green tea. Structurally, EGCG contains eight free hydroxyl groups responsible for its bioactivities properties [2]. Nowadays, the consumption of green tea among the youth has increased in folds, providing numerous benefits to human health [3–5]. Polyphenols represent therapeutic agents against various symptoms. EGCG has inhibitory functions in multiple diseases that arise due to abnormal changes or modifications with an effect as anti-inflammatory properties and antibacterial effects that appear within its effective range. In green tea, EGCG accounts for about 59% of the total catechins and exhibits a broad spectrum of biological activities, such as oral disease linked with microbes. EGCG plays crucial roles against pathogenic microorganisms, such as several Gram-positive and Gram-negative bacteria, and a wide range of anti-infective drugs [6, 7]. Studies have been conducted that proved its role in anti-oxidant, anti-cancer, anti-diabetic, anti-inflammatory, anti-microbial, anti-oxidant, anti-diabetic, neuroprotective activities, anti-cancer, anti-inflammatory, anti-microbial, etc. [8]. Thus, therapeutic advantages from EGCG utilization were observed in oral infections, cancer, obesity, diabetes, neurodegenerative, and inflammatory diseases (Fig. 1).

 EGCG ameliorates oral health by blocking the deterioration of periodontitis dentin erosion and defending oral mucosa against oral tumor cells. Dose-dependent mechanisms for flora-related diseases such as caries and periodontal EGCG have been found to be highly effective. High doses of EGCG act as bactericidal, i.e., kill bacteria by destroying their structures, whereas low doses exert antibacterial effects by reducing virulence, which often leads to bacterial membrane disruption and growth inhibition [9].

EGCG is a promising therapeutic molecule to prevent neurodegenerative diseases (NDs). It has effective antioxidant and anti-inflammatory effects and subsequent therapeutic potential [10, 11]. EGCG can modulate several targets associated with the pathogenesis of several chronic diseases [12–14]. EGCG is promising for promoting healthy aging by recovering the morphologic and functional modifications that happen in a naturally aging brain, suppressing cognitive dysfunction and diminishing oxidative injury in the brain. EGCG possesses neuroprotective effects against several neural injuries [15].

The neuroprotective effects of EGCG were reported in Alzheimer's (AD) and Parkinson's disease (PD) [10, 13]. EGCG has the potential to attenuate cell death, repress ROS accumulation and free intracellular calcium, modify signalling cascades, and consequently decrease oxidative stress. However, in the case of AD, ROS accumulation leads to β-amyloid degradation and repression in the phosphorylation of tau protein [16]. EGCG, with its amyloid precursor protein (APP) processing ability, offers an alternative strategy for AD prevention. Studies have been performed on different cell lines, including MC65, EOC 13.31, SweAPP N2a, and N2a/APP695, proving its anti-neuroinflammatory capacity by inhibiting microglia-induced cytotoxicity [17].

EGCG is a well-explored phytochemical with numerous health benefits that can improve the human lifestyle [18–21]. Its clinical application remains limited due to its poor physicochemical stability and low oral bioavailability. While EGCG exhibits favourable and strong advantages against biochemical and molecular-related diseases, its effectiveness in clinical settings does not fully match that of first-line chemotherapy agents. There is still a certain gap that prevents the real potential of EGCG.

This review focuses on biochemical metabolism, and physiological role in humans. Here, we aimed to compile and present the latest research on biological activities, including the antioxidant, anti-inflammatory, anti-microbial, and neuroprotective properties of EGCG. We further explored the underlying mechanism by which EGCG modulates various cellular signalling pathways, such as those involved in inflammation, apoptosis, and epigenetic modifications, to exert its therapeutic effects. In addition, we summarize the recent findings on the molecular mechanisms and therapeutic effects of EGCG in oral-related and neurological diseases. We discuss the potential of EGCG as a promising therapeutic drug for treating these conditions.

## Biological and pharmacological effects of EGCG

The biological action of EGCG lies in its phenolic hydroxyl groups with molecular structures that are implicated in food production. EGCG has a protecive effect on inflammation due to its antioxidant properties [22, 23]. It prevents cellular oxidation and inhibits cell-free radical damage [24–26]. EGCG attenuates oxidative breakdown under elevated temperatures and alkali states and accelerates the degradation rate with the increase in temperature and oxidation concentration [27]. The effects of EGCG could be highly synergistic when combined with other catechins and thus metabolically stimulated to form stronger and more efficient bioactive compounds [28, 29].

A detailed analysis of the biological effects of polyphenolic compounds present in green tea has shown the difference in pharmacokinetics activity in the individual compounds. It was experimentally proven that intake of 1.5 mM of green tea elevates the level of both ((-)-epicatechin-3-gallate) EGC and EGCG, but the variation is observed in the duration of half-life. For instance, EGC plasma level rises quickly with a low elimination half-time of 1.7 h, whereas EGCG levels rise slowly with a higher half-time of 3.9 h [30, 31].

EGCG is absorbed in the intestine when ingested orally, but its bioavailability has always been a question. Due to its oxidation, efflux, and metabolism, there is a low availability in the gut area. Microbiota present in the gut region deconjugate and degrade EGCG. Studies have even found that EGCG undergoes metabolism before absorption. With the help of gut microbes such as *Enterobacter aerogenes*, *Klebsiella pneumoniae* subspecies EGCG breaks down to EGC and gallic acid [32]. Later, EGC degrades to 5-(3,5-dihydroxyphenyl)−4-hydroxyvaleric acid, which is the main metabolite of EGCG. The absorbed compound is 5-(3’,5’-dihydroxyphenyl)-γ-valerolactone and its glucuronide form is the major urinary metabolite.

The consumption of EGCG has shown various physiological and pharmacological health benefits [33]. EGCG is one of the oldest polyphenols that have been experimented with to test its efficiency on bacteria. EGCG has a potential effect on the growth of *Staphylococcus aureus*, especially methicillin-resistant *Streptococcus* and *E. coli* [34]. EGCG has shown anti bacterial - activity against a heterogeneity of Gram-positive and Gram-negative pathogens, viruses, fungi, and prions. Hence, it is known to be a prospective anti-infective agent. It has antifungal action against human-pathogenic yeast [35, 36].

**Fig. 2:**
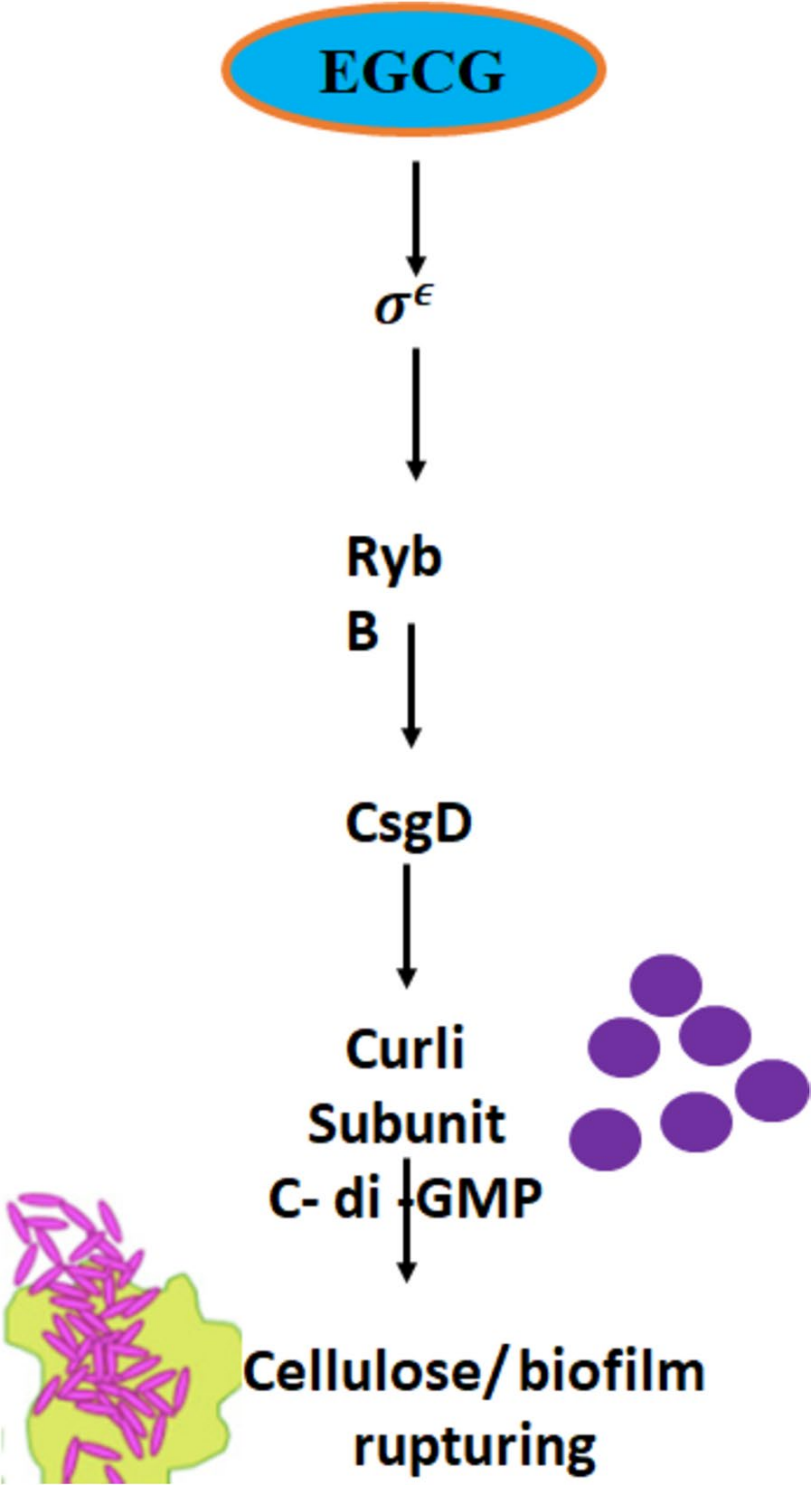
Antimicrobial Action of EGCG through Biofilm Disruption. Showing the antimicrobial mechanism of EGCG, focusing on its ability to disrupt biofilm formation. EGCG targets the σϵ regulatory pathway, which plays a crucial role in bacterial membrane adhesion. By interfering with the production of curli subunits and cyclic di-GMP (c-di-GMP), both essential for biofilm stability and bacterial adherence, EGCG effectively impairs biofilm formation. This disruption weakens bacterial colonization and enhances susceptibility to antimicrobial treatments, highlighting the potential of EGCG as a powerful agent in combating bacterial infections and biofilm-associated resistance

 EGCG inhibits enoyl-acyl carrier protein reductase (ENR) in *Plasmodium falciparum*. The mechanism of inhibition is believed to involve a slow-tight binding mechanism. This means that EGCG binds to the enzyme slowly but forms a very stable complex, effectively inhibiting its activity. Inhibiting ENR would disrupt the fatty acid biosynthesis pathway, which is essential for the survival of parasite. This could lead to reduced parasite viability and potentially prevent or treat malaria infections. Targeting ENR represents a novel approach to combating malaria, which is becoming increasingly resistant to traditional antimalarial drugs [37]. Studies have revealed the mechanism by which EGCG inhibits the occurrence of bacterial outbursts in the body. EGCG interacts with σ, thus modulating the activity of RybB and CsgD genes. This interaction downstream disrupts the biofilm found by the bacteria. This is achieved by alternating curli subunit and c-di-GMP required for membrane formation [38], as described in Fig. 2.

 EGCG induces changes in the proteome of *Microcystis aeruginosa* under stress conditions. It inhibits carbon and nitrogen assimilation along with chlorophyll synthesis and cell division. In contrast, stress response proteins such as superoxide dismutase and glutaredoxin were found to be upregulated. The observed proteomic changes provide insight into the molecular mechanism of the inhibitory activity of EGCG on *M. aeruginosa* [39].

Experimental evidence has proven the relationship between EGCG and exercise and their effect on the physiology of AD. EGCG and exercise, either separately or in combination, are applied to a 2-month mouse, with the impact of a 4-month wheel-running exercise attenuating or lowering the formation of soluble Aβ1–42 levels in the cortex and hippocampus [40]. Also, EGCG enhanced the effects when formulated as dual-drug loaded PEGylated PLGA nanoparticles [41, 42]. However, oral administration of EGCG/AA NPs in mice affected EGCG gathering in all main organs, such as the brain [43]. EGCG might prevent specific biomedical vital molecular targets, including antiapoptotic Bcl-2 proteins and vascular endothelial growth factor (VEGFR) signaling [28, 44–47]. Pharmacokinetic examination accomplished in human exhibits shows that the physiologically pertinent serum concentrations of EGCG could be in the elevated nanomolar range.

EGCG protects against Aβ-mediated cytotoxicity via the Akt pathway activation [16]. The neuroprotective results against Aβ-mediated neuronal cell death have been generated via its capability to scavenge ROS efficiently. The oral administration of EGCG has identified considerable development in spatial cognitive learning capability. EGCG can reduce Aβ and tau toxicity, explaining its promise for the obstacle of AD. EGCG has shown considerable inhibitory results against oxidative stress-mediated cell death. Hence, EGCG is a therapeutic drug in the management of PD [48, 49].

The anti-oxidation process by EGCG is of great importance to human health care [50]. EGCG mitigates the harmful effects of locally administered bupivacaine, a commonly used for epidural anaesthesia and nerve blocks, by suppressing bupivacaine-induced ROS production in neuroblastoma cells. Briefly, EGCG attenuates bupivacaine-induced ROS generation in neuroblastoma cells, protecting against its toxicity [51]. Structurally, the presence of phenol rings in the EGCG acts as a hunter of free radicals and traps the electrons that inhibit the formation of ROS. This causes a decrease in the damage caused by oxidative stress [52–56]. EGCG is an essential compound that received much recognition for its numerous health benefits because it reduces inflammation and helps to prevent heart and brain disease, assisting in weight loss [52].

EGCG prevents stimulated oxidative stress and neurotoxicity, and EC diminishes Aβ-fibril creation. EGCG can control the proteolytic processing of APP, suggesting that green tea polyphenols may be potential therapeutic agents for PD and AD [16]. A marked reduction in β- and γ-secretase function and BACE1 and APP expression was observed [57]. Despite the encouraging effects of pre-clinical examines, a translational gap exists between fundamental invention and clinical application [58]. Multiple studies in the brains of AD patients revealed a decline of PKC€ action in the membrane fraction.

 EGCG acts as a potential inhibitor for the dual-specificity tyrosine phosphorylation-regulated kinase 1A (DYRK1A). DYRK1A overexpression induces morphological defects and impairment in the individuals, leading to Down syndrome, Pick's disease, and AD. EGCG displays appreciable inhibition of DYRK1A even at high concentrations (360 mg/kg). Since the stability is low, structurally modified EGCG shows non-competitive properties. Differing from other published inhibitors, EGCG acts as a non-competitive inhibitor [59]. It revealed that prenatal exposure prevents the over-expression of DYRK1A in the brain. EGCG acts as a competitive inhibitor at the ATP-binding site when DYRK1A induces K465R mutation. Interestingly, the K465R mutation in DYRK1A changes the mode of inhibition of EGCG from a non-competitive to a competitive inhibitor at the ATP site. Although EGCG showed promise as a therapeutic agent (Table 1), its clinical application was limited by its low absorption rate and susceptibility to degradation.

**Table 1:** Target proteins of EGCG

S. No	Target protein	Associated disease	Affinity	Reference
1	Enoyl-acyl carrier protein reductase	*Plasmodium falciparum*	8 nM	[60]
2	3-hydroxyacyl-[acyl-carrier-protein] dehydratase	*Microcystis aeruginosa*	30 nM	[39]
3	Ser/Thr protein kinase mTOR	Oxidative stress	320 nM	[61]
4	DYRK1A	Down syndrome	330 nM	[59]
5	Bcl2	Anti-cancerous	335 nM	[62]
6	NAD Kinase	Lung cancer	3 nM	[63, 64]
7	Cannabinoid receptor 1	Cancer	4 nM	[65, 66]
8	Disintegrin and metalloproteinase domain 17	Cancer	4 nM	[67]
9	Peptidyl- prolyl cis–trans isomerase	Breast Cancer	4 nM	[68]
10	Amyloid-beta precursor protein	Alzheimer's disease	4.60E + 3 nM	[69]

## Molecular mechanisms of EGCG activity

This section provides insights into the intricate interactions of EGCG with key cellular targets, shedding light on its multifaceted therapeutic potential. Antioxidant properties of EGCG are central to its role in reducing oxidative stress and mitigating neuro-traumatization, particularly in neurodegenerative diseases. By scavenging ROS, EGCG helps maintain cellular homeostasis and protects neurons from oxidative damage. Additionally, EGCG induces apoptosis in cancer cells by modulating several pro-apoptotic and anti-apoptotic proteins, effectively promoting programmed cell death in abnormal cells while preserving healthy tissue.

### EGCG reduces ROS oxidative stress

EGCG inhibits cellular mechanisms and tissue oxidative injury by preventing pro-oxidant enzymes. EGCG, having high lipophilic features, illustrated a better affinity for free radicals than the unchanged EGCG [70]. EGCG has been reported as a potent antioxidant and possesses beneficial effects in oxidative stress-related diseases [71].

A study [72] demonstrated the anti-oxidative capabilities of EGCG in H_2_O_2_-mediated oxidative stress of myocardial ischemia injury [73]. The levels of superoxide dismutase (SOD) and glutathione peroxidize (GPx) activity ameliorates with the treatment of H2O2-induced control biopsies with EGCG at the concentration of (50 µg/ml) for 24 h. It has also been observed that when H2O2-induced cervical cancer biopsies treated with EGCG (50 µg/ml) suppress the activity of SOD and GPx by 38.54% and 57.04%, respectively. Similar results were obtained with HeLa cells. Improvement in SOD and GPx activity by co-culture of EGCG indicates it to be an effective natural antioxidant combating ROS generation [74]. Thus, it can be rightfully said that EGCG imparts an inhibitory effect and cancer chemo-preventative agent on the activity of many enzymes and metabolic pathways.

EGG modulates the pathway, which directly inhibits cytokines inflammation and formation of ROS in uric acid-induced human umbilical vein endothelial cells [75, 76]. EGCG reduces the mRNA levels of Notch1, Hey1, and Hes1 in a dose-dependent manner, leading to the inhibition of cancer growth. Additionally, EGCG suppresses Notch promoter activity, further contributing to its anticancer effects. In vivo studies demonstrated a significant reduction in both tumor growth and Notch1 expression in mice injected with EGCG. Similar findings were observed in EGCG-treated colorectal cancer cells, where decreased levels of Hes1 and Notch2 were reported. Notably, EGCG was also found to cleave Notch1 in 5-fluorouracil-resistant colorectal cancer cells, indicating its potential role in overcoming drug resistance [77].

**Fig. 3:**
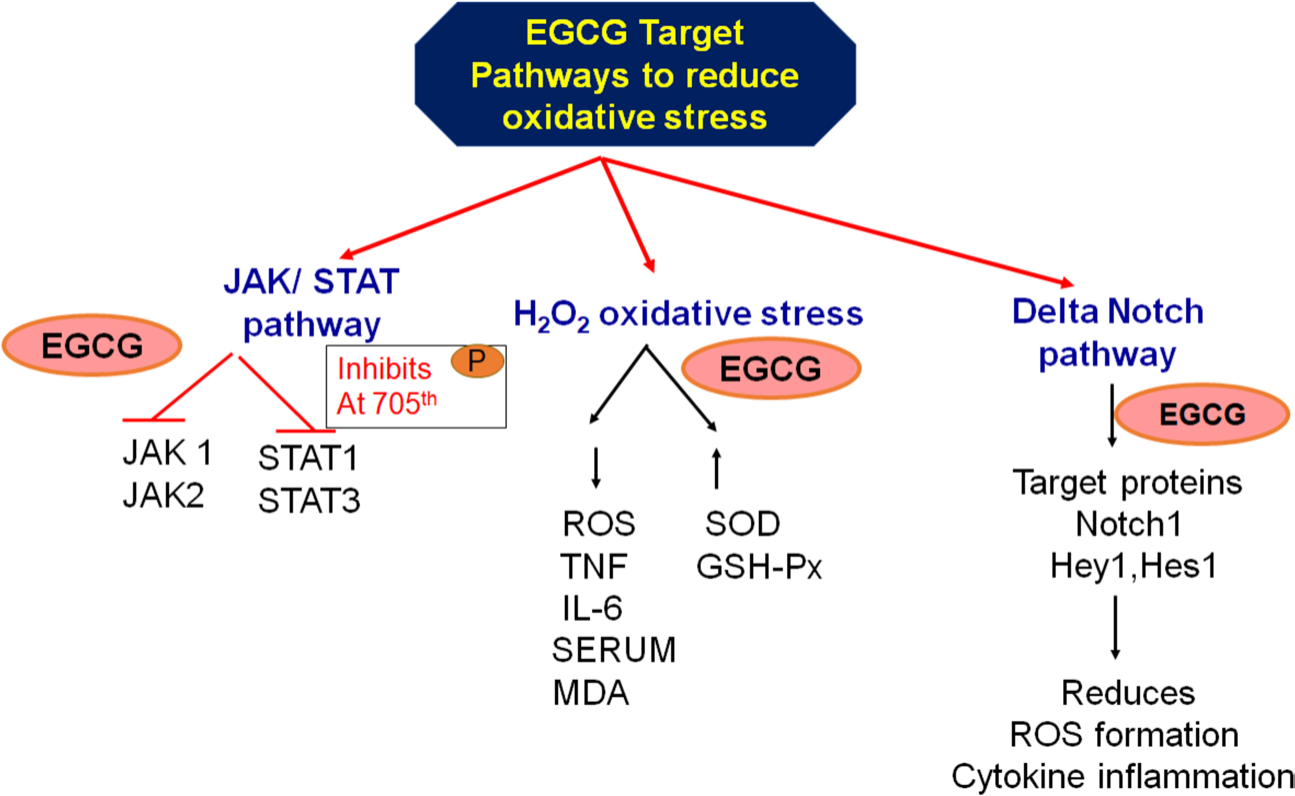
EGCG-Mediated Pathways Targeting Oxidative Stress. Showing the diverse molecular pathways through which EGCG mitigates oxidative stress. EGCG inhibits the phosphorylation of STAT3, thereby disrupting the JAK/STAT signaling pathway, which is crucial for cell proliferation and survival. Additionally, EGCG targets key proteins in the Delta-Notch pathway, including Notch, HEY, and HES1, further influencing cellular differentiation and survival mechanisms

 EGCG significantly improves the survival rate of human umbilical vein endothelial cells exposed to H_2_O_2_-induced oxidative stress [61]. EGCG was found to upregulate key autophagy-related proteins, including Atg5, Atg7, LC3 II/I, and the Atg5–Atg12 complex. It also inhibits the mTOR signaling pathway, further promoting EGCG-induced autophagy **(**Fig. 3**).** In addition, EGCG disrupts the Janus kinase (JAK)/signal transducer and activator of transcription (STAT) signaling pathway at multiple stages by inhibiting STAT phosphorylation, thereby preventing its translocation to the nucleus [78]. Specifically, EGCG inhibits the phosphorylation of the 705th tyrosine residue of STAT, and protein kinase C delta (PKC-delta), effectively blocking STAT1 activity. Moreover, EGCG suppresses the activity of interferon-gamma (IFNγ), JAK1, JAK2, and STAT1/STAT3. Interestingly, EGCG-mono-palmitate, a derivative of EGCG, activate the Src homology 2 domain-containing tyrosine phosphatase-1 (SHP-1) enzyme, which reduced the phosphorylated levels of BCR-ABL and STAT3 in human chronic myeloid leukemia cells [79].

EGCG has shown protective effects when administrated to patients with contrast-induced nephropathy. It normalized kidney function markers, such as serum creatinine and blood urea nitrogen. It is responsible for improving renal tissue health, as indicated by histological analysis. Metabolic physiology showed reduced cell death and oxidative stress within the kidneys and alleviated inflammation in the renal tissue. EGCG-loaded chitosan-casino phosphopeptide nanoparticles show free radical scavenging activity [80].

Furthermore, EGCG decreased both the expression and secretion levels of pro-inflammatory cytokine genes, including TNF-α, IL-1β, and IL-6. EGCG also downregulated the expressions of α-SMA, fibronectin, mast cell trypsin, and chymotrypsin, as well as TGF-β1, CTGF, and PAI-1 [81] as an emerging potential phytocompound EGCG has come upon with its recently discovered role in fibrosis. Due to its anti-inflammatory and anti-proliferative role, EGCG promotes wound healing and prevents scar formation. This is achieved by blocking the pathways of VEGF, CTGF, and TGF-β1. It downregulates the formation of skin scars by reducing the production of COL-1 [82]. EGCG may be effective in treating skin scars by decreasing blood flow and skin thickness, reducing mast cell activity and angiogenesis while also enhancing heme oxygenase-1 levels, increasing M2 macrophage counts, and boosting elastin, antioxidant activity, and hydration [81].

### EGCG reduces traumatic brain injury

EGCG prevents traumatic brain injury (TBI)-mediated IL-1β and TNF-α mRNA in mice brains [83]. EGCG considerably diminished the levels of malondialdehyde (MDA), superoxide dismutase (SOD), and glutathione peroxidase (GSH-PX) after TBI. EGCG diminished TBI-mediated NADPH oxidase activation by inhibiting p47 translocation to the plasma membrane [55, 84]. Preclinical trials reflect the therapeutic potential of EGCG in suppressing the occurrence of secondary brain damage after TBI. The intake of EGCG observes a reduction of brain infarction and oedema in rodent models. EGCG preserves blood–brain barrier integrity through the modulation of tight junction proteins, limiting the entry of blood-derived substances into brain parenchyma. It inhibits the activation of microglial, reducing TNF-α, IL-1β, and IL-6 and alters the NF-κB pathway in the injured rat brain. Additionally, it remarkably reduces neutrophil infiltration and cytokine pro-inflammation [85].

Therapeutic benefits of green tea consumption were reported in inflammatory and NDs [86]. EGCG effectively inhibited IL-8 in epithelial cells. IL-8 is responsible for the recruitment of neutrophils and supports the formation of ROS. EGCG acts as an antioxidant by inhibiting the phosphorylation of IκB to prevent IL-1β-induced NF-κB activation [87]. EGCG inhibited IL-1β-mediated expression of iNOS and COX-2 by inhibiting proteasomal degradation of IκBα [88]. EGCG prevents the binding of AP1 to DNA via IL-1β-mediated phosphorylation of c-Jun, thereby downregulating IL-8 and TNF-α progression. Evidence emerged that supports the inhibition of CD4 + T cells, promotes IκB-α, and reduces ROS formation in neurons by EGCG. EGCG prevents the binding of AP1 to DNA via IL-1β-mediated phosphorylation of c-Jun, thereby downregulating IL-8 and TNF-α progression [89].

EGCG acts as a neuroprotective agent against Aβ-induced oxidative stress by increasing the antioxidants in BV2 microglial cells [90]. EGCG reduces the NF-κB signaling activation, obstructing the formation of cytokines and VEGF in U373MG cells [91]. EGCG represses Aβ-induced neuroinflammatory reactions of microglia, including TNFα, IL-1β, and iNOS, and enhances the levels of antioxidants [92]. It repressed Aβ-induced NF-κB and MAPK pathways, including JNK and p38 [93–96]. EGCG blocks inflammatory and oxidative stress markers via the regulation of nuclear receptor PPARγ in N2a/APP695 cells [97].

### EGCG induces apoptosis: anti-proliferation

EGCG, combined with chemotherapeutic drugs, experimentally showed higher sensitivity against tumor cells. At the same time, the anti-inflammatory and antioxidant effects of EGCG reduce the negative impact caused by the chemotherapy. Clinically, the nanomodification of EGCG significantly increases anti-tumor activity. One such pathway illustrated with doxorubicin (DOX). EGCG enhances anti-tumor activity by inhibiting the activity of carbonyl reductase 1 (CBR1) and P-gp in the liver cells. Here, EGCG binds to CBR1 and inhibits the conversion of daunorubicinol (DNROL) to DOX, enhancing antitumor activity and inducing cardiotoxicity [98].

**Fig. 4:**
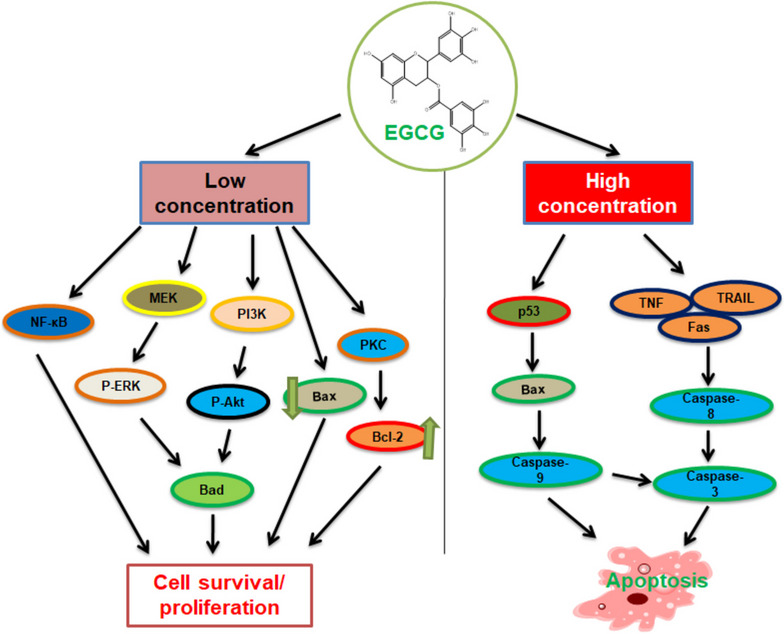
Impact of EGCG on tumor-mediated signaling cascades through apoptosis. EGCG blocks signaling cascade activation and promotes apoptosis. Outline the promising gene targets engaged in anti- and pro-apoptotic activities of low and high EGCG concentration. This effect could be attained via the increased regulation of p53 expression. EGCG enhances the ratio of Bax/Bcl-2 and activates apoptosis

Combining EGCG and sulforaphane with cisplatin significantly increases its effectiveness against both sensitive and resistant ovarian cancer cells. This combination treatment reduces cell survival, accelerates cell death (apoptosis), and halts cell division at the G2/M phase in a time- and dose-dependent manner [99]. EGCG reduced EGFR phosphorylation, suppressed AKT signaling in breast cancer cells, and amplified raloxifene-induced apoptosis [100]. In mice, combined EGCG with tamoxifen leads to a decrease in tumor volume by 71% and tumor weight by 80% by inhibiting mTOR and EGFR expression [101] (Fig. 4).

EGCG administration not only extended the lifespan of lethally irradiated mice but also mitigated radiation-induced damage to the intestinal mucosa. Treatment with EGCG significantly increased the population of Lgr5 + intestinal stem cells (ISCs) and their proliferating Ki67 + progeny while simultaneously reducing radiation-induced DNA damage and apoptosis. Moreover, EGCG effectively lowered ROS levels and activated the Nrf2 transcription factor, which in turn upregulated antioxidant proteins such as Slc7A11, HO-1, and GPX4. Consequently, EGCG acts as a protective agent against radiation-induced injury by scavenging ROS and inhibiting both apoptosis and ferroptosis via the Nrf2 signaling pathway [102].

### EGCG induces cellular signaling and epigenetic modification

EGCG regulates the cell cycle by modulating cyclin-dependent kinases (CDKs). It inhibits the activity of CDK1 and CDK2 and activates cyclin-dependent protein kinase inhibitors such as p17, p21, and p16 [103]. EGCG exerts growth inhibitor effort on human cancer cell lines without affecting normal cells of the body. The effect is dose-dependent, inhibiting cell growth, G0/G1 phase arrest, and DNA damage. Such physiological role indices apoptosis in many cancer cell lines. Molecular docking studies have revealed CDK-6 as the better ligand for EGCG with binding energy − 12.70 and docking energy − 11.40 kcal/mol, as compared to other CDK-6 inhibitors. This study was confirmed with experimental immunoblot assay analysis, which proved that EGCG is an inhibitor of CDK-6 [104, 105].

The role of EGCG in epigenetic modification has always been appreciated. Sjögren’s syndrome (SS) is an autoimmune disease particularly affecting the exocrine glands. There has been a correlation between the syndrome and EGCG, which is responsible for altering the gene expression of related molecules. Such alterations lead to ameliorated salivary gland damage. The mechanism remains the same, including the reduction in ROS activity and accumulation, thereby inhibiting ROS mediating water aqua channel 5. Eventually, EGCG is responsible for increasing the saliva flow [106].

EGCG can inhibit DNA methyltransferases, the enzymes responsible for adding methyl groups to DNA [107]. This inhibition can lead to the demethylation of certain gene promoters, potentially activating genes that were previously silenced. In addition, EGCG has been observed to affect histone acetylation by inhibiting histone deacetylases. Increased histone acetylation typically leads to a more relaxed chromatin structure, which enhances gene transcription. Furthermore, EGCG can influence the expression of microRNAs, which are small non-coding RNAs that regulate gene expression post-transcriptionally. Changes in miRNA levels can affect various cellular processes and contribute to therapeutic effects of EGCG [108].

## EGCG Therapeutic potential in specific human diseases

EGCG is a promising therapeutic agent reported for its diverse biological activities, including antioxidant, anti-inflammatory, and anticancer properties. Many of these interactions require EGCG concentrations far higher than those obtainable through regular green tea consumption or standard green tea extract supplements. Despite this limitation, recent well-conducted clinical trials have demonstrated the efficacy of green tea extracts and purified EGCG products in treating specific conditions. This section focuses on clinically relevant studies, highlighting recent advancements in EGCG-based therapies for diseases. Additionally, this review explores EGCG’s mechanisms of action, examining the existing evidence supporting its therapeutic potential in addressing human diseases.

### Oral health and dentistry

Gingivitis and periodontitis are inflammatory mouth infections in which gums turn red and swollen and sometimes bleed. EGCG helps reduce periodontal disease development by inhibiting the growth of *Porphyromonas gingivalis, Prevotellani crescent*, and *Prevotella* intermedia. These bacteria are engaged in periodontal tissues, causing periodontitis. The efficiency of catechins defends the *gingival* epithelium against the invasion of *Porphyromonas gingivalis,* a potent cause of periodontal disease. Treatment with EGCG blocks the functioning of the Matrix Metalloproteinase-9 (MMP-9) factor and supports osteoclasts in periodontal illness. MMP-2, 8, and 9 present in the dentine region of the oral cavity are responsible for the degradation of tooth decay. EGCG was reported as an MMP inhibitor [109, 110].

Acrolein was found to prevent *gingival* fibroblasts, usually because of cell disruptions and growth. Hence, this might increase several inflammatory situations in the oral cavity, which could cause gingival and periodontal disease. EGCG may decrease the toxicity of acrolein. Therefore, green tea/EGCG has been confirmed as a wonder drug for oral health [111]. Due to inadequate diets among the youth, the uptake of large amounts of carbohydrates and fermented sugars leads to the accumulation of acid-producing microbiota. Among all microbiota, *S. mutans, Lactobacillus,* and *Actinomyces* viscous are dominant and highly implicated in the development of dental caries.

 A high dose of EGCG kills bacterial structure whereas a low dose is effective in anti-bacterial nature by inhibiting virulence factors. In either dose, EGCG leads to destruction in the biofilm growth. Such efficacy is prominent against oral *Candida*. A high dose induces damage to mitochondrial membranes and uncoupling of oxidative phosphorylation. EGCG when combined with other drugs, shows a higher potency towards drug-resistant strains. EGCG is directly involved in the inactivation of oral viruses such as HPV, HSV, and other viral proteins [112].

Tea leaves are rich in fluoride, which improves dental health. Dental caries are induced by oral microflora. Microbial dysbiosis containing Gram‑positive and Gram-negative aerobic and anaerobic bacteria affects the progression of cariogenic dental plaque [112–115]. EGCG inhibits sugar transport and acid formation via lactate dehydrogenase [116]. EGCG is known as a competitive inhibitor of NADPH. In addition, EGCG inhibits the NADPH oxidase translocation and ROS production [117]. Consuming catechins daily efficiently decreases dental caries, and using EGCG/green tea mouthwash diminishes the acidity of saliva and prevents bacterial colonization. The study enhanced salivary pH with EGCG/green tea and decreased dental caries [118, 119].

EGCG acts as the main compound in green tea that was reported to inhibit the growth and virulence factor of microbes of oral pathogens. Though the mechanism is unclear, it has been validated that EGCG is responsible for reducing volatile sulfur compounds by suppressing the *mgl* gene. *Mgl* gene is encoded for enzymes L-methionine-α-deamino-γ-mercaptomethane-lyase, responsible for methyl mercaptan (CH3SH) production by oral anaerobes. EGCG inhibits the growth of *P. gingivalis* and reduces CH3SH production and *mgl* gene expression [112]. The reaction has been carried out by introducing a methyl sulfonyl group into the B-ring of EGCG. A methylation reaction is set to attach the orthoquinone site via oxidation, which facilitates reduction in halitosis. Hence, green tea helps to lower the oral odor [114].

*Solobacterium moorei* is an anaerobic bacterium grouped under a volatile sulfide compound (VSC) associated with halitosis. Upon performing a microplate dilution assay, EGCG was found to show anti-bacterial activity against *S. moorei*. EGCG inhibits the growth of *S. moorei*, with MIC values of 250 μg/ml [120]. Through transmission electron microscopy and a permeabilization assay, it was found that EGCG is responsible for targeting bacterial cell membranes and attenuating their integrity. Similar results were obtained when an analysis was performed on colonization properties. It was found that EGCG significantly reduces colonization and adherence to oral epithelial cells. In addition, EGCG at ½ MIC significantly decreased the β-galactosidase gene expression [120].

### Cancer

#### Oral cancer

EGCG inhibits cell cycle progression and modulates signalling pathways that cause cancer. Its therapeutic potential has been effective against oral cancer. EGCG imparts the G1 arrest of tumor cells, and its treatment significantly enhances caspase-7, 9, and −3 activity along with increased expression of Bax [121]. Indirectly, EGCG is involved in caspases-induced apoptosis.

**Fig. 5:**
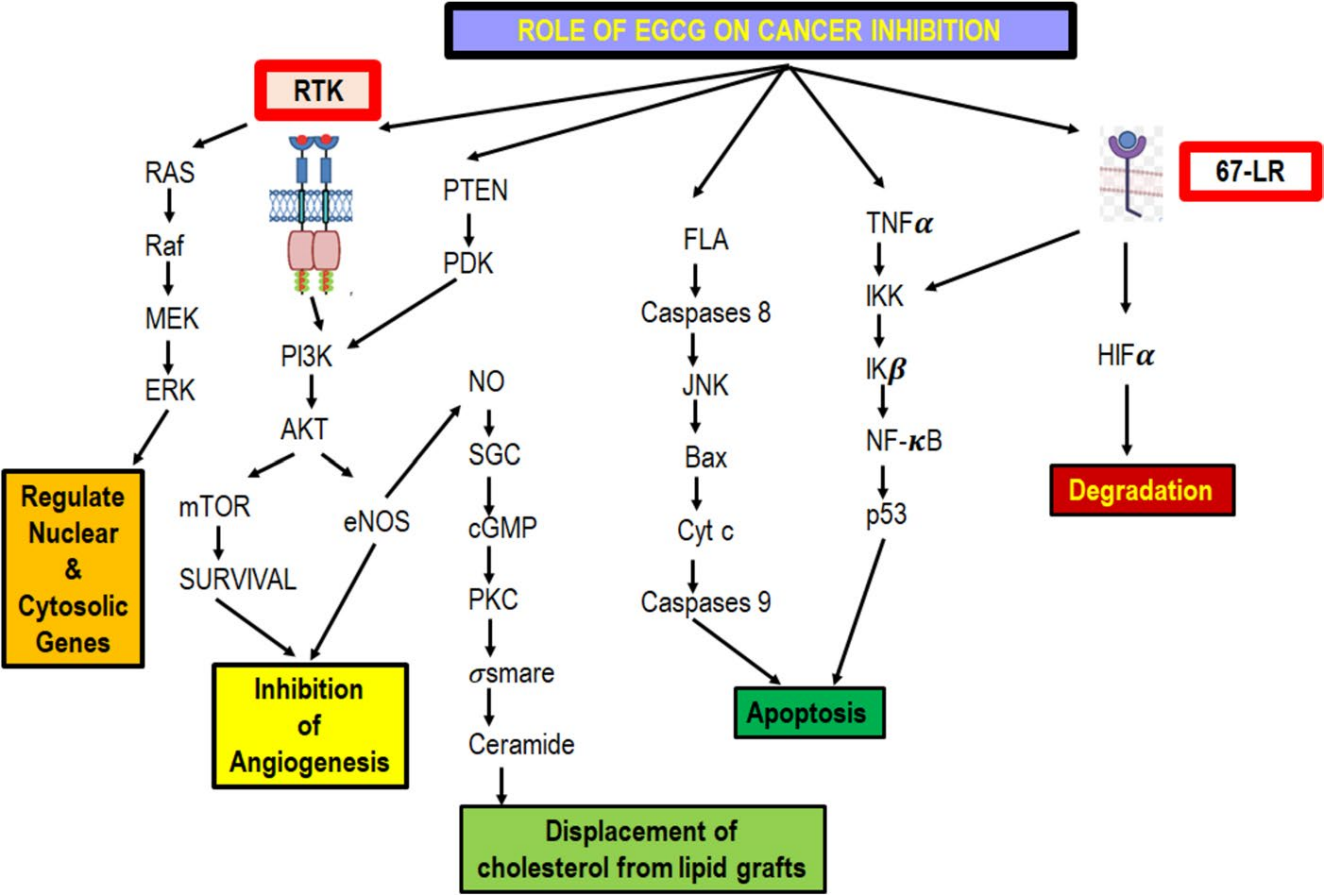
Role of EGCG in inhibition of cancer growth**.** EGCG acts as an anti-cancer agent either by responding through the RTK pathway or by binding to its 67 -ligand-receptor (67-LR). RTK method mediates via RAS pathway regulating nuclear and cytosolic genes. RTK also alter PI3K-AKT pathway thereby inhibiting angiogenesis. EGCG regulates the process of apoptosis via FLA and TNFα

EGCG causes cell death in cancerous cells by modulation of several signalling cascades**.** It diminishes cell proliferation via suppressed Akt, NF-κB, EGFR, and MAPK/ERK1/2 pathways [47]. EGCG acts as a potent drug for tumor chemoprevention capability for cancer transformation of oral premalignant lesions, tumor proliferation blockage, and cell death initiation (Fig. 5).

MMP-9 is associated with oral tumour development [122, 123]. EGCG blocks the invasion and migration of human oral tumour cells by downregulating the MMP 2 and 9 [124] (Table 2). By regulating MMP, metastasis is also prevented. A partial decrease in angiogenesis was observed in VEGF receptor phosphorylation in oral tumours [5]. The effects of EGCG in hindering HGF-mediated proliferation of oral cancers have been reported. EGCG blocks HGF-induced Met phosphorylation and the expression of MMP-9 and −2 [125, 126]. It causes an inhibitory effect on cell motility, migration, and adhesion [127]. The outcomes of green tea/EGCG hinder the development of premalignant lesions for the tumor [128].

**Table 2:** Effects of EGCG on oral diseases

Study Design	Results/Effects	Ref
In vitro	Inhibition of the planktonic growth and the biofilm formation	[129, 130]
In vitro	Decrease of *S. mutans* EPS production	[131, 132]
In vitro	Protected keratinocytes against the TNF- -mediated breakdown of barrier integrity	[133, 134]
In vitro	Prevented bacterial adhesion and *F. nucleatum*-mediated hemolysis	[135, 136]
In vitro	Blocked *S. moorei* growth and bacterial adherence Declined of the biofilm formation Repression of bacterial-galactosidase activity	[135, 137]
In vitro* and *In vivo	Inhibition of p-focal adhesion kinase (p-FAK), p-Src, snail-1, and vimentin	[127, 138]
In vitro	Reduced expressions of MMP-2, MMP-9, and uPA in a dose-dependent way	[124, 139]
In vitro* and *In vivo	Decrease the toxicity of acrolein	[111]
In vitro	Inactivation of oral viruses such as HPV, HSV and other viral proteins	[140]
In vitro	Inhibits the NADPH oxidase translocation and ROS production	[141]
In vitro	Inhibits the growth of *P. gingivalis* and reduces CH3SH production and *mgl* gene expression	[112]
In vitro	Decreased the β-galactosidase gene expression	[142]

#### Breast cancer

EGCG has demonstrated a significant potential in the prevention of various types of cancer. Studies revealed that in a concentration-dependent manner, EGCG shows antioxidant or pro-oxidant properties. Such characteristics endure EGCG to block cell cycle progression and modulate signaling pathways during cancer prognosis. EGCG inhibits the transpiration of VEGF by inducing apoptosis and negatively modulates metastasis . Similar results were obtained from in vivo studies on xenograft animal models [143]. 

EGCG interferes with estrogen receptor signaling, which is critical in estrogen receptor-positive breast cancers, potentially reducing the proliferation of these cancer cells. It inhibits the formation of new blood vessels (angiogenesis), which is essential for tumour growth and metastasis in breast cancer. EGCG enhances the efficacy of common breast cancer treatments, such as tamoxifen, by sensitizing cancer cells to these therapies.

Even the overall expression of cyclins (Cyclin D, Cyclin E, CDK 4, CDK 1, and PCNA) was down-regulated in time-dependent increasing concertation of the EGCG-treated group as compared to the untreated control group by Western blot analysis that blocked the G1 phase of the cell cycle [47]. Experiments on nude mice induced with human MDA-MB-231 breast cancer cells and treated with EGCG show an effective reduction in tumour incidence [144].

EGCG has demonstrated prominent inhibition in the growth of cancer stem cells (CSCs) when used alone and when used in combination with other chemotherapeutic drugs. It reduces the resistance of CSCs and intracellular efflux. Moreover, EGCG increases the drug concentration in CSCs by inhibiting the activity of the intracellular transporter gene family, ABC transporters, at minimal concentration [145]. EGCG does not affect the ABC transporter in glioma CSCs but is able to downregulate P-glycoprotein, thus reducing colony formation and the migration of tumour cells [146]. EGCG at low concentration inhibits colony formation in pancreatic ductal carcinoma, but when used in combination with PDE3A inhibitor trequinsin, it lowers the expression of proteins FOXO3 and CD44 [147]. Upregulation of cGMP expression is observed, which showed a significant reduction in colony formation [148].

#### Salivary gland tumors

EGCG inhibits β1 integrin, thereby reducing the expression of MMP-2 and MMP-9, thus providing molecular evidence for the inhibitory effect of EGCG on salivary gland cancer metastasis [149]. Studies have reported that EGCG inhibits cell proliferation and expression of EGFR, downregulates Bcl-2, and upregulates Bax;, therefore, inducing apoptosis of adenoid cystic carcinoma [150]. EGCG mitigates inflammatory damage to the salivary glands by suppressing inflammatory cytokines and reducing oxidative stress. Moreover, it promotes the growth and repair of salivary gland tissue. Additionally, EGCG inhibits the metastasis of salivary gland cancer by downregulating MMP proteins [151, 152].

#### Prostate cancer

EGCG administration leads to the downregulation of cyclin D and cyclin E, which are involved in G1/S progression. This indicates that EGCG induces G1 phase arrest in prostate carcinoma cells. Similar role was observed in breast cancers suggesting chemo-preventiverole of EGCG [153]. Treatment with ECGC on LNCaP prostate cancer cell lines reduces cell proliferation. There was increased expression of androgen receptor and prostate-specific antigens on the cancer cell lines [154]. EGCG induces apoptosis in prostate cancer cells. It activates pro-apoptotic proteins and inhibits anti-apoptotic proteins, leading to the selective killing of cancer cells while sparing normal cells [155]. EGCG can cause cell cycle arrest in prostate cancer cells, particularly at the G1 phase, preventing cells from progressing to the S phase and thereby inhibiting tumor growth. Prostate cancer growth is often driven by androgen receptor signalling. EGCG downregulates androgen receptor expression and function, reducing the proliferation of androgen-dependent prostate cancer cells. In addition, EGCGinhibits the expression of MMP enzymes that degrade the extracellular matrix and facilitate cancer cell invasion and metastasis. By inhibiting MMPs, EGCG reduces the metastatic potential of prostate cancer cells [149].

#### Others

EGCG exerts anti-cancer effects across various cancer types through multiple mechanisms, including the induction of apoptosis, inhibition of cell proliferation, suppression of metastasis, and modulation of epigenetic changes [58, 156]. Its ability to target specific signaling pathways and enhance the effectiveness of existing treatments for cancer prevention and therapy [157]. EGCG diminished the gefitinib-induced overexpression of CYP1A1, CYP1B1, EGFR, cyclin D1, p-Akt (Ser473), and survivin at both the transcriptional and translational levels. EGCG upregulated phosphorylation p53 at Ser15, thereby enhancing gefitinib's therapeutic efficacy against benzo[a]pyrene-induced lung cancer [158].

EGCG inhibit lung cancer cell growth by causing cell cycle arrest and promoting apoptosis. It reduces the metastatic potential of lung cancer cells by downregulating the expression of MMPs and other factors involved in cell migration and invasion [33]. By reducing oxidative stress, EGCG decreases the risk of lung cancer initiation and progression, especially in smokers and individuals exposed to environmental carcinogens [159].

Due to its numerous health benefits, EGCG has been increasingly used in the treatment of acute and chronic respiratory diseases. EGCG affects tumour initiation and progression by downregulating angiogenesis and metastasis. Its involvement is remarkable in increasing tumor suppressor genes, apoptosis, neoplastic cells, and cell cycle arrest. EGCG downregulates (Intercellular adhesion molecule) ICAM-1 expression and the counts of neutrophils and eosinophils in the bronchoalveolar lavage fluid (BALF) in human pulmonary alveolar epithelial cells [160].

An evident correlation was observed between EGCG and Nonalcoholic fatty liver disease (NAFLD). EGCG effectively ameliorated NAFLD disorder and its phenotypic [161]. It inhibits intestinal barrier and inflammations. This is achieved by increasing the concentration of gut microbes, including short-chain fatty acid (SCFA) producers such as gram-negative *Lactobacillus*. An increase in SCFA level inhibits the TLR4/NF-κB pathway, thereby alleviating liver inflammation. Thus, it suggests that the role of EGCG on gut microbiota is to reduce the inflammation in hepatocytes [141].

EGCG targets survival pathways such as PI3K/Akt and NF-κB, which are often upregulated in pancreatic cancer [162]. This leads to reduced cell proliferation and increased apoptosis. EGCG enhances the sensitivity of pancreatic cancer cells to chemotherapy, potentially overcoming resistance to conventional treatments [163]. EGCG inhibits the metastatic spread of pancreatic cancer cells by downregulating enzymes and signalling molecules involved in invasion and migration (Fig. 5).

EGCG was reported as a noncompetitive inhibitor of NAD kinase with a *K*i of 3.28 ± 0.32 μM. SPR analysis revealed a* K*_d_ of 1.78 ± 1.15 μM, while HDX-MS indicated binding at non-substrate sites on NADK [63]. EGCG selectively inhibited the growth of KRAS-mutant lung cancer cells without affecting KRAS wild-type cells [164].

### Cardiovascular diseases

#### Atherosclerosis

Atherosclerosis is a disease of the arteries caused by endothelial dysfunction, inflammatory vascular cells, and lipid accumulation. EGCG-treated mice showed a 23% decline in aortic weights and cholesterol as well as TG [165]. Studies exhibited a considerable decrease in LDL-C, which was examined in EGCG-treated subjects. Therefore, these outcomes recommend that EGCG has the potential to block the progression of CVD and hypertension via an important decrease in LDL-C [166, 167]. Studies revealed that EGCG acts as an inhibitor of HDAC1, leading to better functioning of the heart. It decreases heart/body weight and the ratio of mtDNA/nDNA. In addition to inhibiting HDAC1, EGCG increases the binding of acetylated H3K9 or H3K14 in the promotor regions of peroxisome proliferator-activated receptor-1α and nuclear respiratory factors [168].

#### Cardiac hypertrophy and heart failure

The protection of cardiac homeostasis comprises hypertrophy and enlargement of heart size, as well as increased protein synthesis, which are the major symptoms of cardiac hypertrophy that generally cause a decline in the heart's capability to pump blood to the organs. The therapeutic potential effect of EGCG in treating hypertension-mediated learning as well as memory impairment is possibly due to its influential antioxidative roles [169]. EGCG is helpful in fighting against aging-mediated cardiac hypertrophy, fibrosis, and cell death [170]. EGCG prevents the development of heart failure in mice via inhibition of myocardial fibrosis and decline of ventricular collagen remodelling [171]. EGCG inhibits NF-κB activation and subsequent TGF overexpression. However, it diminished the expression of fibronectin and the proliferation of rat cardiac fibroblasts [165, 172]. The treatment of EGCG drastically recovered cardiac diastolic role via up-regulating cTnI via preventing histone deacetylase 1/3 expression. EGCG can contribute to the hindrance of cardiac diastolic dysfunction [173].

### Neurodegenerative disorders

An effective and well-mentioned role of EGCG is to reduce the formation and accumulation of ROS production and increase pro-apoptotic markers in various tissues. EGCG imparts neuroprotective effects and widens neuro-rescue actions; thus, EGCG shows neuroprotective effects. Some human studies have revealed a dose-dependent relationship between EGCG intake and neuro-related disorders such as AD and PD [10, 174].

PD is a neurodegenerative disorder associated with the accumulation of Fe, oxidative stress, and inflammation. It is distinguished by nigrostriatal degeneration, which may involve α-synuclein (αS) aggregation, dysregulation of redox metal homeostasis, and neurotoxicity. However, tea polyphenols play a vital function, halting the progression of PD development. Tea polyphenols can directly obstruct the aggregation of the αS protein and alter signaling cascades in animal models [175].

EGCG prevents αS aggregation and interferes with PD development. The aggregation has reduced via EGCG in a concentration-dependent way, as revealed through a lack of thioflavin T binding [176, 177]. αS aggregates have been identified via incubation with A-Syn-HiLyte488. This binding has been inhibited via EGCG [176]. The αS amino acid positions that are interrelated with EGCG have been identified on peptide membranes. EGCG attaches to αS through unstable hydrophobic interfaces. EGCG might be a promising altering drug of αS aggregates for the management of PD and other α-synucleinopathies [176].

EGCG post-treatment significantly rescued 1-methyl-4-phenyl-1,2,3,6-tetrahydropyridine (MPTP)-induced neurotoxicity by increasing the rotational latency and increasing dopamine [178–181]. EGCG has antioxidant and iron-chelating properties. EGCG blocked MPTP-mediated neurotoxicity by raising the locomotor activity, positive neurons, and striatal dopamine (DA) concentrations. A decline in TH action with iron, EGCG inhibited MPTP-induced neurodegeneration and rescued dopaminergic neurons after MPTP treatment in iron excess condition [182–184].

**Fig. 6:**
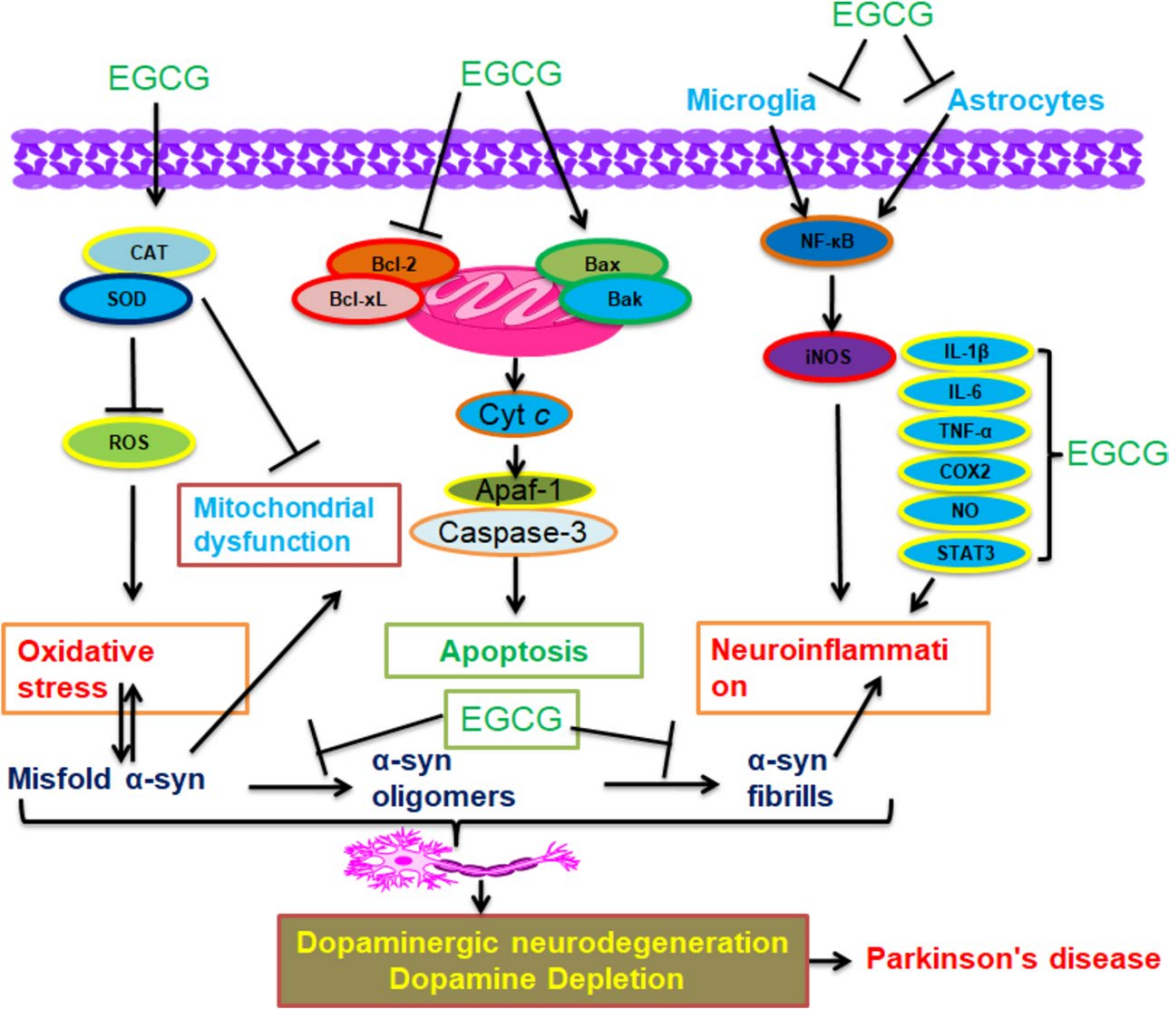
The potential neuroprotective effects of EGCG in PD**.** The mechanisms of EGCG exert neuroprotective advantages. EGCG can prevent protein misfolding, neuronal apoptosis, oxidative stress, and neuroinflammatory responses

The neuroprotective functions of EGCG were reported in PD models. Because of its multi-targeted activities, EGCG might have the potential to be a new drug for the treatment of PD and for preventing neurodegeneration. The molecular mechanism by which EGCG exerts neuroprotective advantages, such as inducing apoptosis and inhibition of inflammation, oxidative stress, modulation of dopamine making, and the aggregation of αS, is described in Fig. 6.

 The main pathological characteristics of AD is amyloid β peptide (Aβ) accumulation in the brain [185–187]. Aβ is believed to be a vital neuroinflammatory stimulus to microglia. Treatment with EGCG has been found to reduce the aggregation of amyloid [16]. Aβ aggregates have been diminished after EGCG treatment in APP transgenic animal models. EGCG controlled (amyloid precursor protein) APP processing and subsequently reduced Aβ deposition [16].

**Fig. 7:**
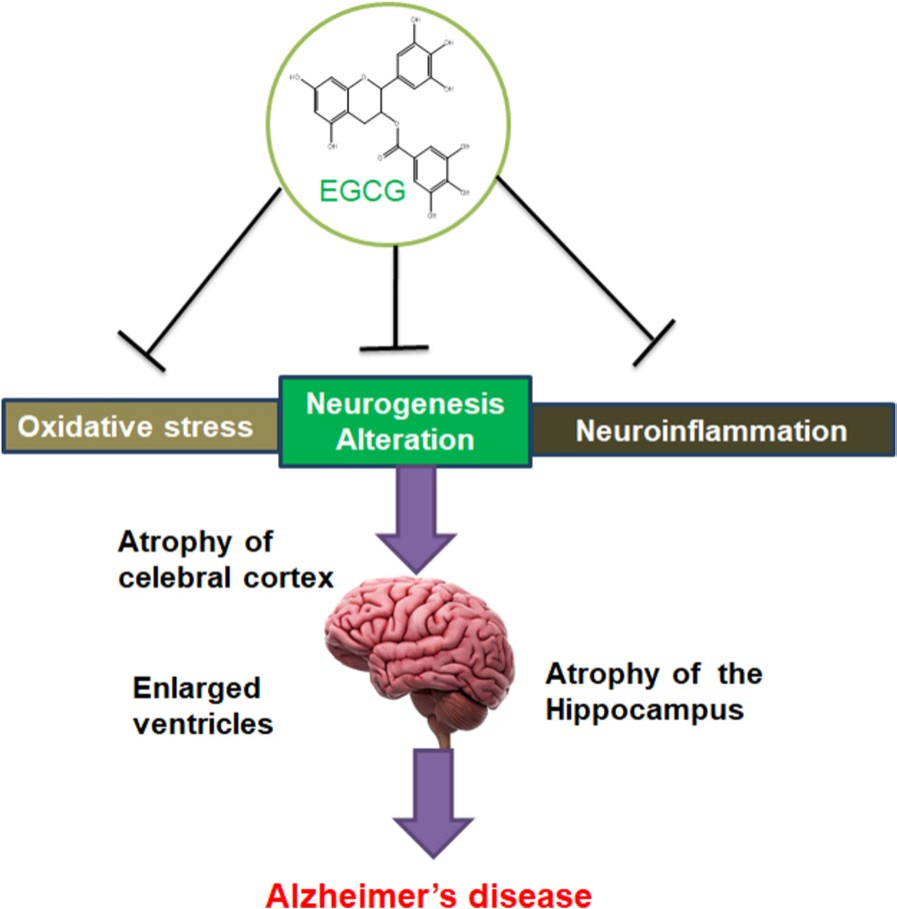
The promising effects of EGCG in AD pathogenesis**.** EGCG participates in a neuroprotective function and is prospectively utilized as a therapeutic drug for managing AD

EGCG is involved in the non-amyloidogenic procedure via stimulating α-secretase cleavage in SweAPP N2a cells. EGCG enhances sAPP-α and diminished Aβ formation in MC65 cells, hence showing the defensive nature of EGCG on brain edema and neuronal injury[16]. EGCG bypassed the blood–brain barrier (BBB) to accomplish the efficient parts of the brain. EGCG is being considered a promising agent for managing NDs [188–190]. The function of EGCG in treating AD has been studied, demonstrating that EGCG participates in a neuroprotective function and is promising to be utilized as a therapeutic drug for treating AD (Fig. 7).

EGCG enhances the production of soluble proteins of AβPP, sAβPPα through PKC-dependent α-secretase activation. Cleavage of AβPP to sAβPPα leads to a non-amyloidogenic secretory pathway accomplished via a putative α-secretase, therefore precluding the formation of Aβ, the last is controlled through the sequential activity of β and γ-secretases [191]. The neuro-protecting results of EGCG against Aβ-mediated neuronal loss and tau toxicity in AD models have been identified in multiple studies. EGCG, with its APP processing ability, provides a hopeful and different strategy for AD prevention [16].

### Metabolic disorders

#### Diabetes mellitus

Metabolic diseases (MDs), diabetes, and obesity are the most common disorders [192]. Diabetes mellitus (DM), one of the most frequent metabolic disorders worldwide, is attributed to hyperglycemia caused by either reduced insulin emission or insulin resistance. Multiple studies have revealed that type 2 diabetes mellitus (T2DM) might stimulate various complications, including diabetic cardiovascular complications, diabetic nephropathy, and neuropathy, which were the main reasons for its mortality and morbidity [193]. The chief pathophysiologic factors that cause T2DM are peripheral insulin resistance and the last devastation of insulin creator pancreatic cells [194, 195].

**Fig. 8:**
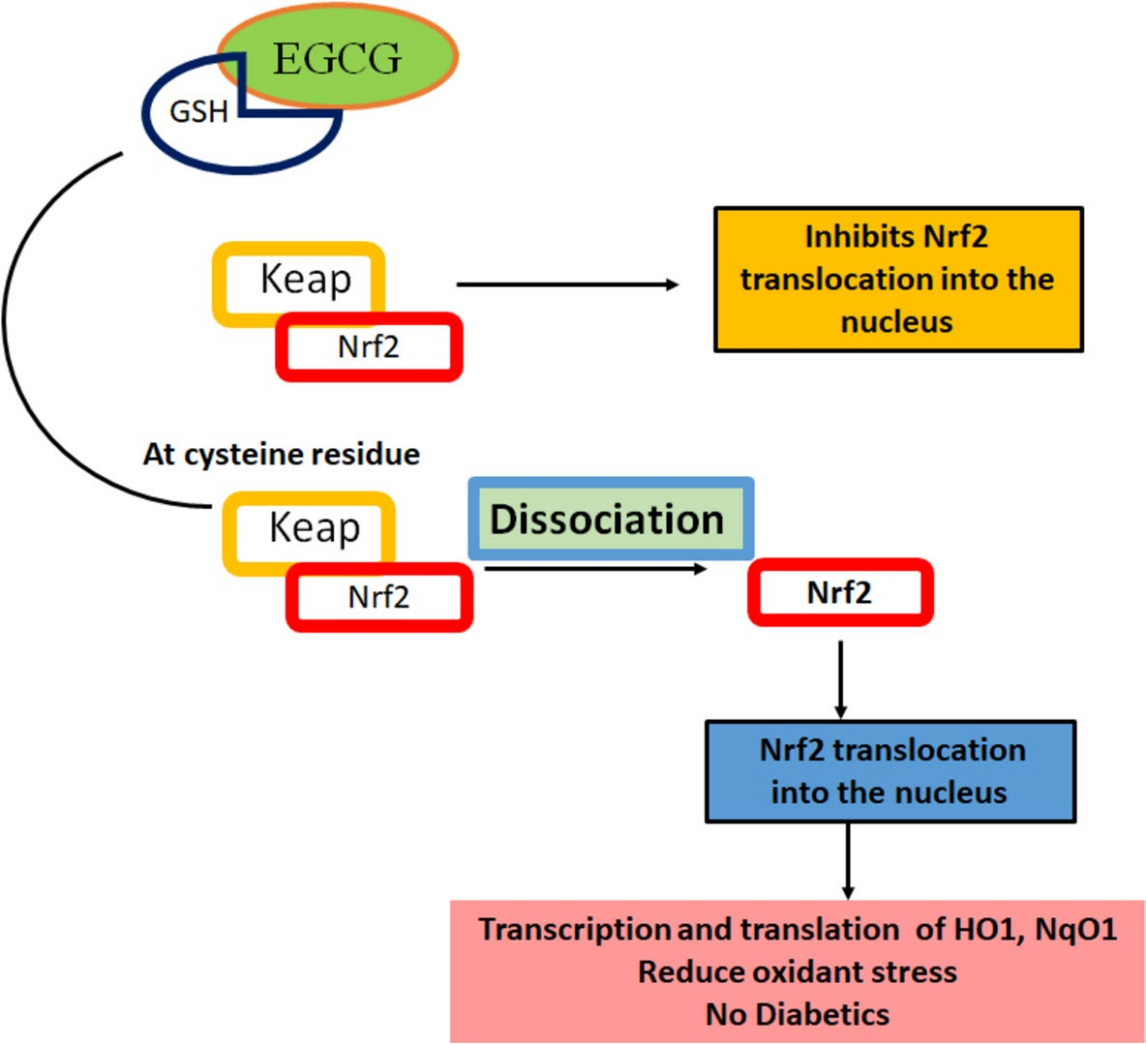
The Role of EGCG in Diabetes Management. Showing the pivotal role of EGCG in diabetes, particularly through its interaction with the KEAP1-Nrf2 signaling pathway. EGCG forms a hybrid complex with glutathione (GSH), which binds to KEAP1, resulting in the dissociation of KEAP1 from Nrf2. This dissociation allows Nrf2 to translocate into the nucleus, where it initiates the transcription of crucial antioxidant proteins, including heme oxygenase-1 (HO-1) and NAD(P)H quinone oxidoreductase 1 (NQO1). These antioxidant proteins play an important role in reducing oxidative stress and inflammation, key contributors to diabetic complications

The antioxidant properties of EGCG have been studied in multiple diseases. However, improving the bioavailability of EGCG via nano-formulation might contribute to a more productive treatment of DM metabolic consequences and vascular complications [196]. Although the mechanisms by which EGCG is related to the onset of DM are still unknown, the possibility might explain the hyperglycemia-induced inflammation (Fig. 8). A report has discussed a case of popular interstitial EGCG composed of MMP-9-bearing cells in a type II DM patient. EGCG improves insulin sensitivity and glycaemic control and drastically diminish serum triglycerides and total cholesterol levels following long-term supplementation [142]. Additionally, EGCG declined triglycerides and considerably enhanced HDL and glucagon-like peptide 1 levels in a randomized, double-blinded, placebo-controlled clinical trial linking patients with T2DMs and lipid abnormalities [197].

#### Obesity

Obesity is predominantly the outcome of a positive energy equilibrium determined by enhanced calorie-enrich food utilization and minimal exercise. It is considered a low-grade systematic inflammation disease. It could react quickly and animatedly to modifications in nutrient excess via adipocyte hypertrophy as well as hyperplasia. Obesity is principally motivated by an inequity between energy intake and expenditure; this modification leads to an improvement of the adipose tissue due to the gathering of lipids that happens in peripheral-related organs [198]. EGCG is shown to have anti-obesity as well as anti-diabetic effects [199]. The promising role of ECGC as an antioxidant and anti-inflammatory in the formation of gut microbiota and favours the formation of bacteria such as *Alloprevotella* and *Muribaculaceae* [200]*.* EGCG enables the reduction of negative regulating bacteria*, Blautia,* hence mining the functions of probiotics to improve intestinal inflammation and treat obesity.

Apart from gut improvement, EGCG shows its prominent role in balancing the level and proportion of gut SCFAs, the expression levels of inflammatory and transcription factors, and alterations in hypothalamic neurotransmitters. This suggests the effect of EGCG on the overall body axis, thereby improving obesity and its induced inflammatory response [201].

Although epidemiological and clinical studies explain the health advantages of EGCG on diabetes and obesity, the mechanisms of its actions are promising based on several laboratory data. EGCG had a considerable outcome on the diminished obesity in body weight gain and decline in epididymal adipose tissue weight, which influenced serum lipid attributes, such as triglyceride and cholesterol [202].

## Conclusions and future prospects

EGCG, a primary polyphenol present as a major composition in green tea, has achieved a significant attantion due to its proven importance in maintaining human health. It has a wide range of biological activities in preventing various disease. EGCG has antioxidant, anti-inflammatory, and anti-microbial properties. These activities are connected to its ability to inhibit the growth of cancer cells, prevent tooth decay, reduce inflammation, and protect neurons from damage. EGCG improves overall health in oral dentistry, including bad breath, oral cancer, tooth decay, and oral cavity inflammation.

While preclinical studies highlight the potential of EGCG in treating various diseases, rigorous clinical trials are needed to establish its efficacy and safety in humans. Future research should focus on determining the optimal dosage, treatment duration, and delivery methods to maximize therapeutic benefits while minimizing potential side effects. Efficient delivery of EGCG to target tissues remains a challenge due to its poor bioavailability and rapid metabolism. Future research could explore advanced delivery systems such as nanoparticles, liposomes, or other innovative drug delivery technologies that enhance stability and bioavailability of EGCG, allowing it to reach specific tissues more effectively.

EGCG modulates various signaling pathways and opens avenue for its use in combination with other therapeutic agents. EGCG could be tailored to target specific signaling pathways unique to certain cancer types. This precision approach may lead to more personalized cancer therapies with higher efficacy. Future studies could investigate the synergistic effects of EGCG when combined with other drugs or natural compounds, potentially enhancing treatment outcomes for conditions like cancer, neurodegenerative diseases, and metabolic disorders.

In addition to advancements in genomics and personalized medicine, there is potential to tailor EGCG-based therapies to individual genetic profiles. Although EGCG is generally considered safe, long-term studies are needed to assess its safety profile, particularly at higher doses or with prolonged use. Investigating potential interactions with other medications and understanding the long-term effects on different organ systems will be crucial for its safe therapeutic application.

EGCG has significant potential as a chemopreventive agent, particularly in individuals at high risk of developing certain cancers. Future studies could explore the role of EGCG in preventing cancer initiation and progression, potentially leading to its use in preventive strategies for at-risk populations. The role of EGCG in neuroprotection, cancer prevention, and oral health, future studies could explore its potential in other areas such as cardiovascular health, diabetes management, and immune system modulation. Expanding the scope of research could uncover new therapeutic applications for EGCG. The future of EGCG in disease therapy lies in its potential to serve as both a preventive agent and a therapeutic adjunct. By developing advanced delivery systems, combining EGCG with existing therapies, and conducting rigorous clinical trials, EGCG could become an integral part of newer treatments.

For EGCG to transition from experimental studies to clinical practice, it must undergo rigorous evaluation . EGCG holds significant promise as a therapeutic agent for various diseases, particularly in neuroprotection, cancer prevention, and oral health. However, realizing its full potential requires continued research, clinical validation, and the development of innovative delivery methods. Overall, the available evidence suggests that EGCG has the potential to be a safe and effective treatment for several oral diseases and neurodegenerative diseases. Hence, more investigation is needed to validate these findings and to develop more effective delivery methods for EGCG.

## Data Availability

All data generated or analyzed during this study are included in this manuscript and are attached to this article.

## Abbreviations

EGCG: Epigallocatechin 3-gallate
AD: Alzheimer's disease
PD: Parkinson's disease
(Aβ): Amyloid β peptide
COX-2: Cyclooxygenase 2
MMP-9: Matrix metalloproteinase-9
NF-κB: Nuclear Factor Kappa B
ERK1/2: Extracellular signal-regulated kinases1/2
H_2_S: Hydrogen sulfide
CH_3_SH: Methyl mercaptan
ROS: Reactive oxygen species
H_2_O_2_: Hydrogen peroxide
VEGF: Vascular endothelial growth factor
NHL: Non-Hodgkin lymphomas
TNF: Tumor necrosis factor
EGFR: Epidermal growth factor receptor
